# Site-specific single point mutation by anthranilic acid in hIAPP_8–37_ enhances anti-amyloidogenic activity†

**DOI:** 10.1039/d0cb00178c

**Published:** 2021-01-15

**Authors:** Sourav Kalita, Sujan Kalita, Ashim Paul, Manisha Shah, Sachin Kumar, Bhubaneswar Mandal

**Affiliations:** Laboratory of Peptide and Amyloid Research, Department of Chemistry, Indian Institute of Technology Guwahati Assam-781039 India bmandal@iitg.ac.in; Department of Biosciences & Bioengineering, Indian Institute of Technology Guwahati Assam-781039 India

## Abstract

Amylin or hIAPP, together with insulin, plays a significant role in glucose metabolism. However, it undergoes β-sheet rich amyloid formation associated with pancreatic β-cell dysfunction leading to type-2 diabetes (T2D). Recent studies suggest that restricting β-sheet formation in it may halt amyloid formation, which may limit the risk for the disease. Several peptide-based inhibitors have been reported to prevent aggregation. However, most of them have limitations, including low binding efficiency, active only at higher doses, poor solubility, and proteolytic degradation. Insertion of non-coded amino acids renders proteolytically stable peptides. We incorporated a structurally rigid β-amino acid, Anthranilic acid (Ant), at different sites within the central hydrophobic region of hIAPP and developed two singly mutated hIAPP_8–37_ peptidomimetics. These peptidomimetics inhibited the amyloid formation of hIAPP substantially even at low concentration, as evident from *in vitro* ThT, CD, FT-IR, TEM, and Congo red staining birefringence results. These peptidomimetics also disrupted the preformed aggregates formed by hIAPP into non-toxic species. These β-amino acid-based peptidomimetics can be an attractive scaffold for therapeutic design towards T2D or other protein misfolding diseases.

## Abstract 2

β-Amino acid based peptidomimetics are attractive scaffolds for therapeutics design towards T2D. They prevent amyloid formation of hIAPP by forming non-fibrillar non-toxic aggregates.

## Graphical abstract

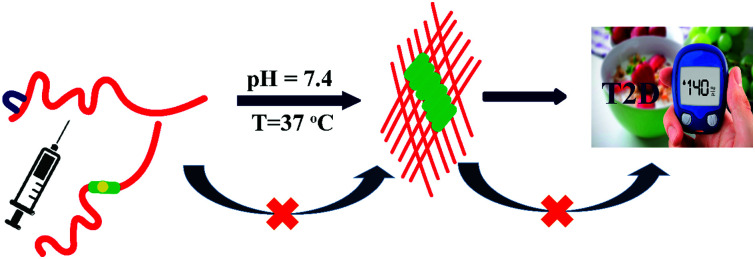

## Introduction

Protein misfolding and amyloid aggregation cause many human diseases, including Alzheimer's disease (AD), Type II Diabetes (T2D), and Parkinson's disease.^1^ Despite the morphological variations, amyloid aggregates causing these diseases are rich in highly ordered cross-β-sheet structures.^2^ Amylin or human Islet Amyloid Polypeptide (hIAPP), co-secreted with insulin from pancreatic β-cell, changes from non-toxic monomers to toxic oligomers at physiological conditions. These toxic oligomers form pores in the β-cell membrane causing β-cell death. This pathogenesis gradually progresses and finally leads to Type II Diabetes.^3,4^ Hence, preventing the amyloidogenic peptide from acquiring a β-sheet rich conformation can become a therapeutic strategy for the inhibition of amyloid formation and uprooting amyloidogenic diseases.^5,6^

Although hIAPP complements insulin in keeping the glucose equanimity by suppressing glucose secretion and regulating gastric emptying, it is highly amyloidogenic.^7^ Development of an effective inhibitor of hIAPP aggregation is a challenging task. Until now, no cure for T2D exists. However, one of the most popular strategies is β-sheet breakers, where one recognition moiety targets the protein of interest, and the activity element averts β-sheet formation. One positive aspect of peptides is their ability to bind a large target surface efficiently and selectively, which is the prerequisite of protein–protein interactions (PPI).^8–10^ This feature of the peptides enables their use as a therapeutic agent, which has grown rapidly over the decades, including metabolic and cardiovascular diseases.^11^

We previously demonstrated the inhibitory efficacy of β-sheet breaker hybrid peptidomimetics (BSBHPs) by inserting one breaker element, isomers of aminobenzoic acid, into the core hydrophobic region of hIAPP (hIAPP_22-27_). We found 2-Aminobenzoic acid or Anthranilic acid (**Ant**) is an efficient breaker element.^12^**Ant** induces conformational restriction in the peptidomimetics due to its structure. In the structure of **Ant**, both the amine group and the carbonyl group are directly connected to the aromatic moiety, constituting a planar structure with a fixed dihedral angle, ∅ = 0°. The structural rigidity of peptides containing **Ant** are significantly enhanced due to the π-stacking ability of the aromatic moiety.^13,14^ Due to its higher structural rigidity, we inserted Anthranilic acid as a β-breaker element within the central hydrophobic region of hIAPP peptide. Furthermore, insertion of **Ant** in peptide sequences favors either a turn or a helix conformation.^15,16^

Earlier, Raleigh *et al.* observed that a single point mutation by proline in hIAPP converts it into a highly effective inhibitor.^7^ Further, a three proline containing analog of hIAPP, with less tendency to aggregate, called Pramlintide (PM), has been approved for clinical applications. However, it also suffers from low solubility, particularly at physiological pH, preventing its co-formulation with insulin.^17^ Moreover, peptides are highly susceptible to proteolytic degradation; but introducing β-amino acids instead of α-amino acids to the peptide sequence decreases the proteolytic degradation.^12,18^

The core hexapeptide, NFGAIL (hIAPP_22–27_), is highly amyloidogenic and a significant driver for hIAPP aggregation. The **Ant** containing small BSBHPs was found useful to inhibit the aggregation of hIAPP and disrupt its preformed fibrillar assemblies at a relatively higher dose (10-fold molar excess). These BSHBPs are stable towards proteolytic degradation due to non-coded β-amino acid **Ant**.^12^ However, these BSBHPs may not effectively bind with the full-length hIAPP due to smaller size and lack of residual interaction. Probably, therefore, a high dose was necessary for the inhibition of aggregation.

**Scheme 1:**
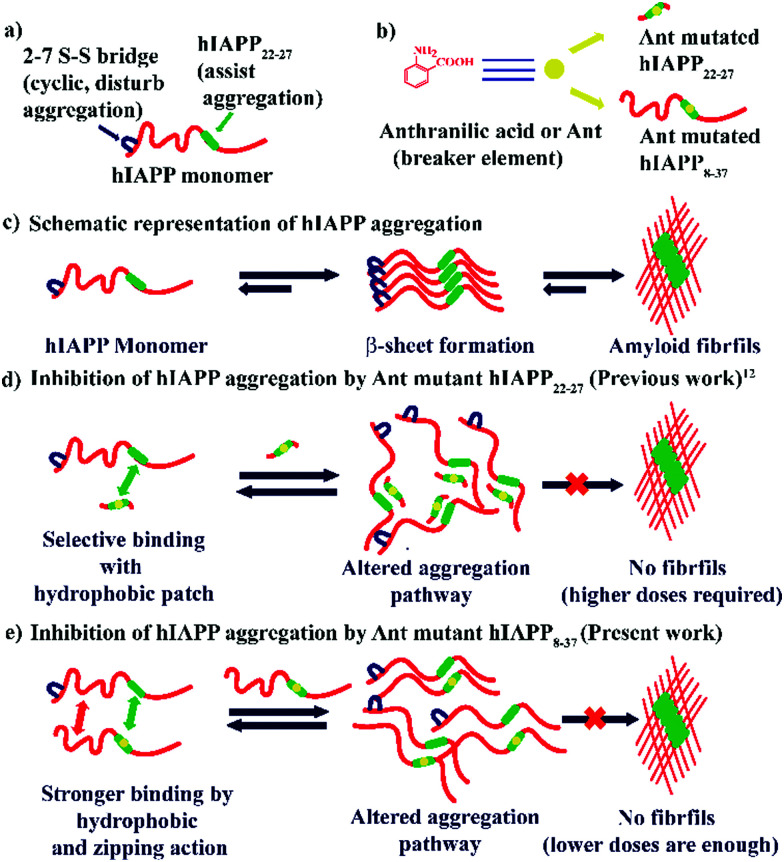
(a) Cartoon representation of hIAPP monomer. (b) Representative structure of **Ant** and **Ant** mutated breaker peptides (c) schematic representation of hIAPP aggregation where it converts from a random coil structure (monomeric form) to amyloid (fibrils). (d) Proposed hypothesis for the inhibition process of amyloid formation by an **Ant** mutated hIAPP_22–27_. (e) Proposed hypothesis for the inhibition of amyloid formation by **Ant** mutated hIAPP_8–37_.

Although the mechanism is not fully understood, it is hypothesized that the inhibitors need to bind with the growing fibrils of the aggregating peptide to inhibit aggregation, and a mutant of the full-length hIAPP may bind more tightly than the small fragment of hIAPP.^7,19^ Therefore, we decided to synthesize a new set of peptidomimetics, comprising of hIAPP_8–37_ with a single point mutation at different positions, which are expected to exhibit inhibitory effect at fewer molar ratios compared to the smaller hIAPP_22–27_ variant (Scheme 1).

## Results and discussion

### Peptide design

For the present study, we synthesized hIAPP_8–37_, without any breaker element (**A**, Table 1), to verify whether it aggregates as much as hIAPP_1–37_ does. The first seven residues of hIAPP are not involved in forming a β-sheet for the conformational restrictions imposed by the disulfide bridge; we, therefore, eliminated that part.^19^ Further, to prove our hypothesis, we synthesized two peptidomimetics by incorporating **Ant** in the sequence of hIAPP_8–37_ at two different positions (at G24 for **B1** and I26 for **B2**). These two positions lie within the core hydrophobic region critical for hIAPP aggregation. Several peptide-based inhibitors are reported using the hIAPP_20–29_ fragment.^12,19–21^ Moreover, proline mutation in this region is partially responsible for rat Amylin's non-amyloidogenic nature.^22^ Therefore, we targeted this specific region of hIAPP_8–37_ to incorporate the breaker elements in the present study. We also synthesized a control breaker peptidomimetic with the same breaker element in the smaller hIAPP_22–27_ fragment (at I26 as **C**).

**Table 1:** Sequences of synthesized polypeptides and peptidomimetics and their functions

Code	Sequence	Functions
**A**	^8^ATQRLANFLVHSSNNFGAILSSTNVGSNT^37^Y	Aggregating
**B1**	^8^ATQRLANFLVHSSNNF**X**AILSSTNVGSNT^37^Y	Breaker
**B2**	^8^ATQRLANFLVHSSNNFGA**X**LSSTNVGSNT^37^Y	Breaker
**C**	^22^NFGA**X**^27^L	Control

As the entire hexapeptide is responsible for forming β-sheet containing amyloid fibril, we may select any one position to incorporate the breaker element inside the peptide sequence. Hence to maintain sequence homology as a standard control breaker peptide, we selected I26 for mutation with **Ant** instead of G24.^12,20,21^

The designed peptidomimetics were synthesized by standard solid-phase peptide synthesis method using Fmoc/*t*-Bu strategy on Rink Amide MBHA resin, purified by reverse-phase HPLC, and characterized by MALDI mass spectrometry (ESI S1–S8, ESI†).^23^ Commercially available wild type hIAPP_1–37_ was used as a native aggregating system for the present study.

### Non-amyloidogenic nature of the synthesized polypeptides and peptidomimetics

We first examined the amyloidogenic nature of the synthesized polypeptides and peptidomimetics by various biophysical tools. They were dissolved in PBS (50 mM, pH 7.4) to obtain a concentration of 40 μM and incubated at 37 °C. After five days, their self-aggregation propensity was monitored by TEM and Congo-red stained birefringence study. Also, the conformational changes were characterized by CD and FTIR analyses.

**Fig. 1:**
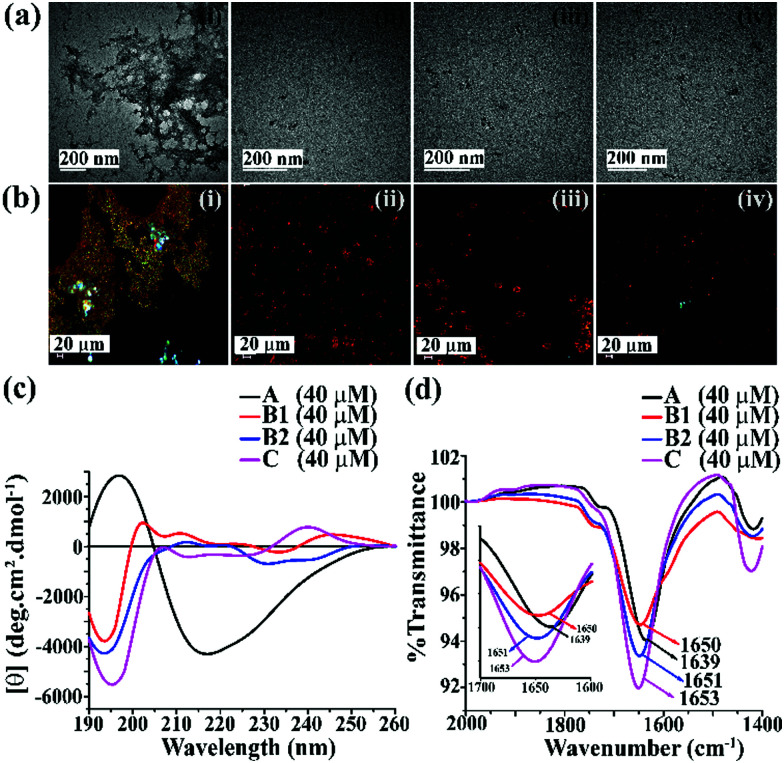
(a) TEM and (b) Congo red-stained birefringence images of **A** (i), **B1** (ii), **B2** (iii), and **C** (iv), respectively. Images were taken after five days of incubation in PBS at pH 7.4 and 37 °C. The scale bar for TEM and Congo red stained birefringence is 200 nm and 20 μm, respectively. (c) CD and (d) FTIR spectra of **A** (black), **B1** (red), **B2** (blue) and **C** (magenta). Spectra were recorded after five days of incubation in PBS at pH 7.4 and 37 °C. A zoom-in inset image for the FTIR spectra has been incorporated in Fig. 2(b).

The appearance of fibrillar assembly under an electron microscope is a characteristic property of amyloid.^24^ Similarly, the presence of green gold birefringence under cross-polarised light upon staining with Congo red is another characteristic feature of amyloid formation.^24^**A** showed a clear fibrillar structure under TEM and green gold birefringence under cross-polarised light (Fig. 1(a and b)); however, no such characteristic appearance was noted for **B1**, **B2**, and **C**, respectively (Fig. 1(a and b)).

After five days of incubation, **A** displayed a positive band at ∼195 nm and a negative band centered at ∼225 nm (black, Fig. 1(c)) in CD, indicating its β-sheet rich conformation. Similarly, we observed a strong amide I band at 1639 cm^−1^ for **A** (black, Fig. 1(d)) in the FTIR profile, which is also a characteristic feature of a β-sheet rich conformation.^24^ In contrast, the other three peptides, **B1**, **B2**, and **C**, did not show any such characteristic features for β-sheet rich conformation in CD and FTIR, suggesting their non-amyloidogenic nature.^15,24^

The above results indicate that **B1**, **B2**, and **C** do not form amyloid under physiological conditions. Nevertheless, **A**, which lacks turn-inducing **Ant** in the backbone, is highly amyloidogenic similar to wild-type hIAPP.

### Inhibition of amyloid formation of hIAPP by the designed peptidomimetics

**Fig. 2:**
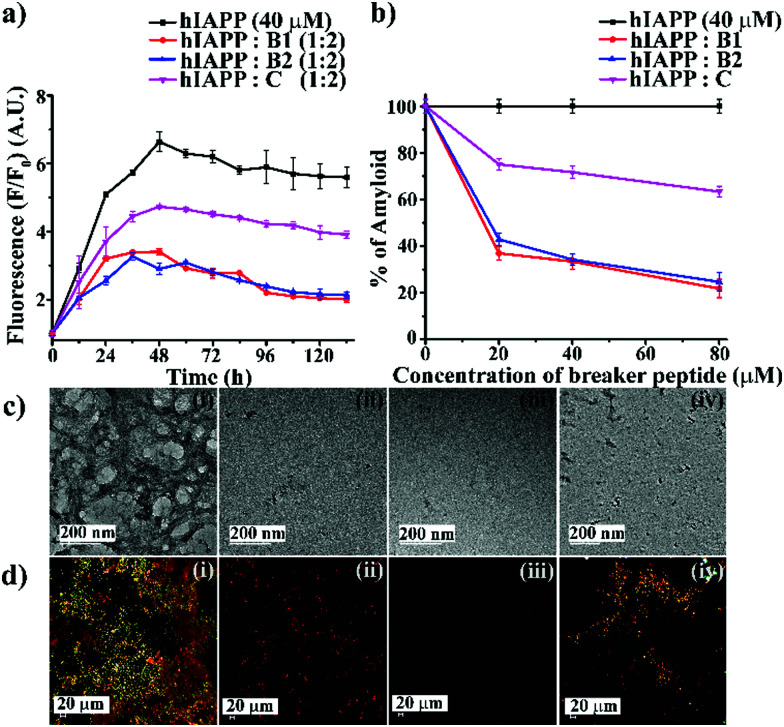
(a) Time-dependent ThT assay of hIAPP (40 μM) in the absence (black) and presence of 2-fold molar excess of **B1** (red), **B2** (blue), and **C** (magenta). (b) Dose-dependent ThT assay of hIAPP (40 μM) in the absence (black) and presence of various molar excess of **B1** (red), **B2** (blue), and **C** (magenta). (c) TEM and (d) Congo-red birefringence images of hIAPP (i) alone and in the presence of 2-fold molar excess of peptidomimetics, **B1** (ii), **B2** (iii) and **C** (iv). The scale bar for TEM and Congo red stained birefringence is 200 nm and 20 μm, respectively.

To investigate the inhibitory efficacy of the single mutant hIAPP_8–37_ and to compare the results with single mutant hIAPP_22–27_ as a negative control, we accomplished various biophysical studies in the absence or presence of synthesized peptidomimetics in different doses (hIAPP : peptidomimetics = 1 : 0.5, 1 : 1, and 1 : 2). We did not include **A** for further studies as it was amyloidogenic. Wild type hIAPP_1–37_ was incubated in the absence or presence of peptidomimetics in PBS (pH 7.4) and 37 °C for seven days. The kinetics of amyloid formation of hIAPP was monitored by time-dependent Thioflavin T (ThT) fluorescence assay, TEM, Congo-red stained birefringence, CD, and FT-IR. In the ThT assay, the amyloid formation is measured by the amount of increment in fluorescence intensity.^24^ The fluorescence intensity of hIAPP in PBS alone increased with time (black, Fig. 2(a)), but that in the presence of 2-fold molar excess of **B1** (red, Fig. 2(a)) and **B2** (blue, Fig. 2(a)) got suppressed significantly (up to ∼75–78%), indicating inhibition of amyloid formation by **B1** and **B2**. On the other hand, the control peptidomimetic **C** (magenta, Fig. 2(a)) inhibited amyloid aggregation, only up to ∼36%. While 0.5-, 1- and 2-fold molar excess of **B1** (red, Fig. 2(b)) exhibited 63%, 66%, and 78% of hIAPP aggregation inhibition, respectively, the same doses of **B2** (blue, Fig. 2(b)) exhibited 57%, 65%, and 75%, respectively. However, **C** (magenta, Fig. 2(b)) exhibited only 24%, 28%, and 36% inhibition, respectively, with the same doses (ESI S9, ESI†). Hence, a dose-dependent effect was noted for all the breaker peptidomimetics.

After seven days of incubation, hIAPP alone exhibited clear fibrillar morphology (Fig. 2(c)-(i)) when viewed under TEM. However, hIAPP, when incubated with a 2-fold molar excess of **B1** (Fig. 2(c)-(ii)) and **B2** (Fig. 2(c)-(iii)), no such fibrils were observed, indicating substantial inhibition of aggregation. On the contrary, in the presence of 2-fold molar excess of **C** (Fig. 2(c)-(iv)), some fibrillar assembly was observed, indicating less efficiency of **C**. Further, hIAPP alone showed green gold birefringence under cross-polarised light, after staining with Congo-red (Fig. 2(d)-(i)), indicating amyloid formation. On the other hand, in the presence of 2-fold molar excess of **B1** (Fig. 2(d)-(ii)) and **B2** (Fig. 2(d)-(iii)), no such green-gold birefringence was observed, which indicated significant inhibition of amyloid aggregation by the peptidomimetics. However, in the presence of **C** (Fig. 2(d)-(iv)), some characteristic green-gold birefringence was observed, implying relatively less efficiency of the same to inhibit amyloid aggregation. After seven days of incubation, hIAPP alone exhibited clear β-sheet rich conformation, as evident from CD and FTIR analyses. But, hIAPP, when incubated with a 2-fold molar excess of **B1**, **B2**, and **C**, such β-sheet conformation was not observed in both CD and FTIR spectra, indicating inhibition of β-sheet formation. Similar results were obtained with 0.5- and 1-fold molar excess of peptidomimetics when co-incubated with hIAPP with relatively less efficiency (ESI S10 and S11, ESI†). All the above results collectively indicate that **B1**, **B2**, and **C** were able to inhibit the aggregation of wild type hIAPP. However, **B1** and **B2** emerged to be better inhibitors than **C**. No significant difference in the inhibition capability of **B1** and **B2** was noted, which implies the position of **Ant** does not alter aggregation inhibition capability significantly. Therefore, we proceeded with **B1** only for other experiments.

### Disruption of preformed amyloids by the designed peptidomimetics

**Fig. 3:**
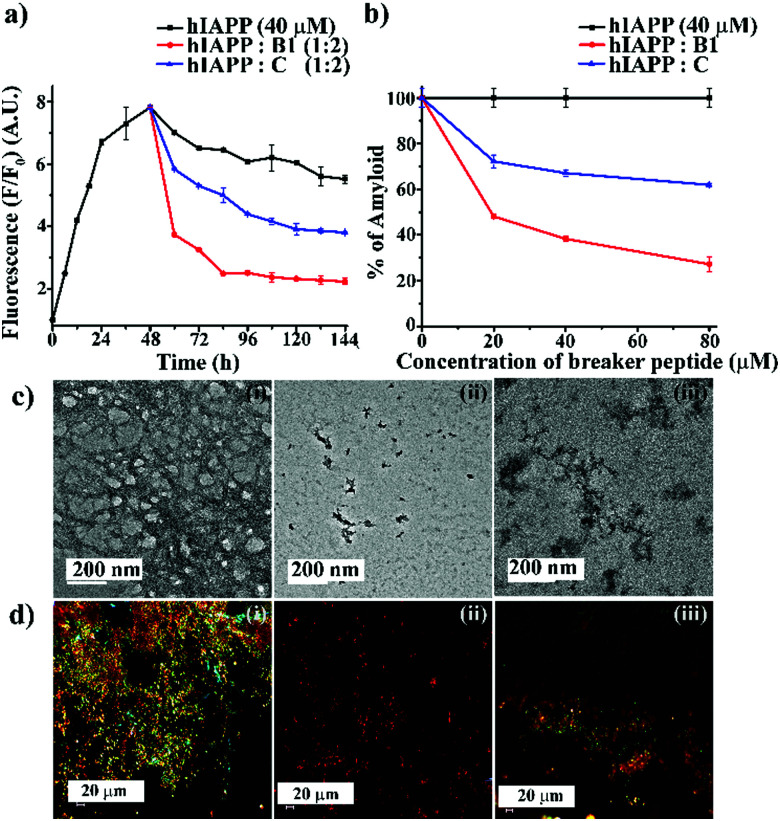
(a) Time-dependent ThT assay of hIAPP (40 μM) in the absence (black) and presence of 2-fold molar excess of **B1** (red) and **C** (blue). (b) Dose-dependent ThT assay of hIAPP (40 μM) in the absence (black) and presence of varied molar excess of **B1** (red) and **C** (blue). (c) TEM and (d) Congo-red birefringence images of hIAPP (i) alone and in the presence of 2-fold molar excess of **B1** (ii) and **C** (iii). The scale bar for TEM and Congo red stained birefringence is 200 nm and 20 μm, respectively.

Next, we investigated the preformed amyloid disrupting capability of **B1**. From the ThT fluorescence assay (black, Fig. 2(a)), it was noted that the fibrillization of hIAPP reaches a plateau at around 45–50 h. Therefore, to carry out the disruption study, we allowed to incubate hIAPP alone in PBS up to 48 h at pH 7.4 and 37 °C followed by the addition of the designed peptidomimetics (**B1** and **C**) into it in various molar ratios (1 : 0.5, 1 : 1 and 1 : 2) like the earlier experiments. The time-dependent ThT fluorescence studies indicated that when hIAPP_1–37_ was incubated alone, the fluorescence intensity increased with time, reached a saturation level, and became almost steady (black, Fig. 3(a)). However, in the presence of the 2-fold molar excess of **B1** (red, Fig. 3(a)), a significant amount of disruption of the preformed fibril was noted after seven days. On the other hand, in the presence of **C** (blue, Fig. 3(a)), the fluorescence intensity got suppressed up to a small extent at the same interval, indicating it is relatively less efficient in disruption in that molar excesses.

From the dose-dependent studies, it was inferred that in the presence of 0.5-, 1- and 2-fold molar excess of **B1** (red, Fig. 3(b)), hIAPP exhibited 51%, 61%, and 72%. In contrast, with the same doses of **C** (blue, Fig. 3(b)), it exhibited 27%, 32%, and 38% of fibril disruption, respectively. Hence, the ability of fibril disruption by the designed peptidomimetics increased in a dose-dependent manner (ESI S12, ESI†).

After seven days of incubation, hIAPP alone (Fig. 3(c)-(i)) showed fibrillar assembly under the electron microscope (TEM). Whereas, in the presence of 2-fold molar excess of **B1** (Fig. 3(c)-(ii)), hIAPP did not show any such fibrillar assembly indicating significant disruption of preformed amyloid. However, a 2-fold molar excess of **C** (Fig. 3(c)-(iii)) could not disrupt the preformed fibril completely. Similarly, hIAPP (Fig. 3(d)-(i)), when incubated alone, exhibited clear green-gold birefringence under the cross-polarised light after staining with Congo-red dye, indicating amyloid formation. But, in the presence of 2-fold molar excess of **B1** (Fig. 3(d)-(ii)), no such characteristic birefringence was observed, indicating significant disruption of preformed amyloid. However, in the presence of the same equivalent of **C** (Fig. 3(d)-(iii)), some green gold birefringence persisted, which indicated incomplete amyloid disruption.

The fibril disrupting-ability was also investigated using CD and FT-IR. When hIAPP was incubated alone for 7 (2 + 5) days at physiological condition, β-sheet conformation was observed in the CD profiles. However, in the presence of 2-fold molar excess of **B1**, a random coil conformation was observed, which indicated disruption of the preformed amyloid of hIAPP.

On the other hand, when **C** was present in 2-fold molar excess, β-sheet rich conformation was observed, indicating inefficiency (ESI S13, ESI†). Similarly, after seven days of incubation, hIAPP alone showed a sharp band at 1634 cm^−1^ in FT-IR spectroscopy, a characteristic amide I band for aggregated β-sheet. Again, when **B1** was present in 2-fold molar excesses, the band shifted to a 1654 cm^−1^, indicating its β-sheet disrupting capability. In the presence of 2-fold molar excess of **C** too, the band shifted to 1648 cm^−1^. The disruption of preformed amyloid improved in a dose-dependent manner (ESI S13 and S14, ESI†). The above systematic studies collectively indicated **B1** was highly efficient in disrupting the preformed amyloid of hIAPP with 2-fold molar excesses only, whereas **C** was significantly less effective at that concentration.

### Vesicle leakage study

The soluble oligomers are more toxic than the full-grown fibrils of hIAPP as they can damage the cell membrane by forming pores into it.^25,26^ Ramamoorthy *et al.* reported that the hIAPP_1–19_ does not form amyloid fibrils but possesses the potency to disrupt artificial lipid vesicles similar to full-length hIAPP and proposed a model for studying membrane disruption by hIAPP and other amyloidogenic peptides.^27,28^ The two-step process commences the cellular membrane disordering by the aggregating peptide *in vitro*. The soluble oligomers bind to the cell membrane first, forming some tiny ion-selective channel-like pores. As the pores formed by the aggregating peptides are unstable, the pores may merge into larger aggregates and convert into fibers during the membrane disruption process. On the other hand, in the next step, the fibril growth of the aggregating peptides may cause non-selective physical membrane disruption *via* a detergent-like process.^27,28^

We noted that both **B1** and **C** disrupted preformed amyloid. To investigate whether they converted the fibrils into membrane-damaging toxic species after disruption, we carried out a dye leakage assay using carboxyfluorescein dye entrapped Large Unilamellar Vesicles (LUVs). LUVs acted as an artificial cell membrane. We prepared five sets of samples to perform the vesicle leakage assay, including the untreated LUVs (without any peptide) as a control. **B1** and **C** were added separately to the preformed fibrillar assemblies of hIAPP, *i.e.*, after 48h of incubation, and co-incubated for an additional five days (168 h = 48 h + 120 h). The different samples prepared for the dye leakage assay was brought up as follows:

Sample 1 – untreated LUVs

Sample 2 – hIAPP (after 5h incubation) + LUVs

Sample 3 – hIAPP (after 7 days incubation) + LUVs

Sample 4 – hIAPP: B1 (1 : 2) + LUVs

Sample 5 – hIAPP: C (1 : 2) + LUVs

**Fig. 4:**
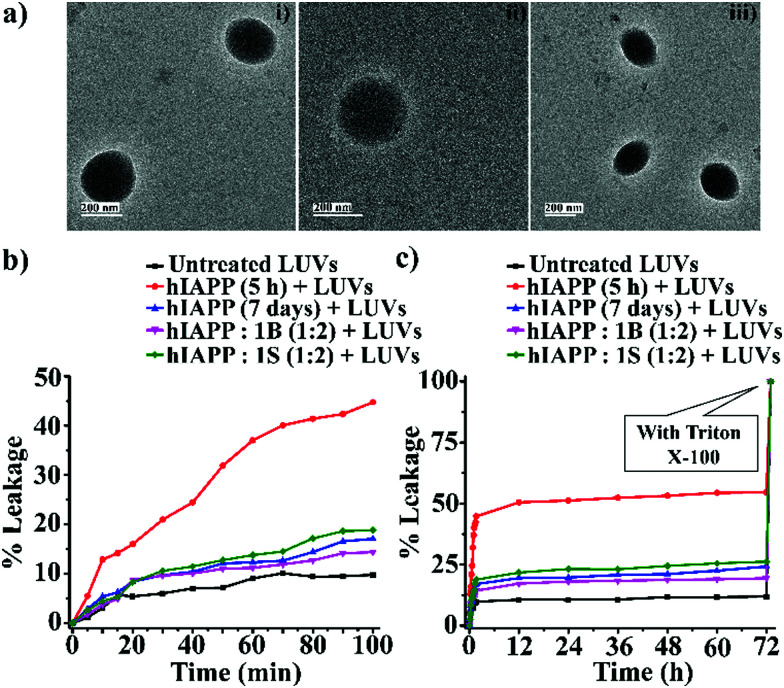
(a) TEM images (i, ii and iii) of the LUVs (1 mM) in PBS buffer (50 mM). Images were captured after the immediate preparation of the vesicles. The scale bar is indicated as 200 nm. The emission of carboxyfluorescein dye showing the effect of hIAPP on the large Unilamellar vesicles (LUVs) with time and % of dye leakage. (b) Dye release from LUVs in the absence and presence of different samples from 0 to 100 min. (c) Dye release from LUVs in the absence and presence of different samples from 0 min to 72 h.

The complete dye leakage was achieved by the addition of Triton X-100 (10 μL) and considered it as total fluorescence. The percentage of dye leakage was calculated as^29^
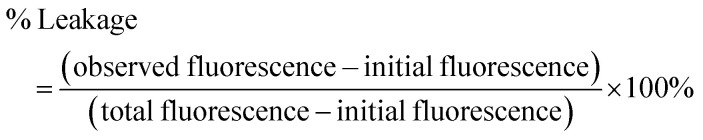
In the dye leakage assay (Fig. 4), we observed a rapid increment in fluorescence intensity until 100 min from LUVs in sample 2 (*i.e.*, with 5 h old hIAPP) that reached a plateau after 12 h, indicating significant dye leakage at the first hours. This dye leakage means the oligomers formed after five hours created pores on the LUVs. Carboxyfluorescein dye leaked from the LUVs, causing an enhancement of fluorescence intensity. Thus such 5 h old oligomers were more membrane damaging, therefore more toxic than the full-grown fibrils obtained after seven days.

**Fig. 5:**
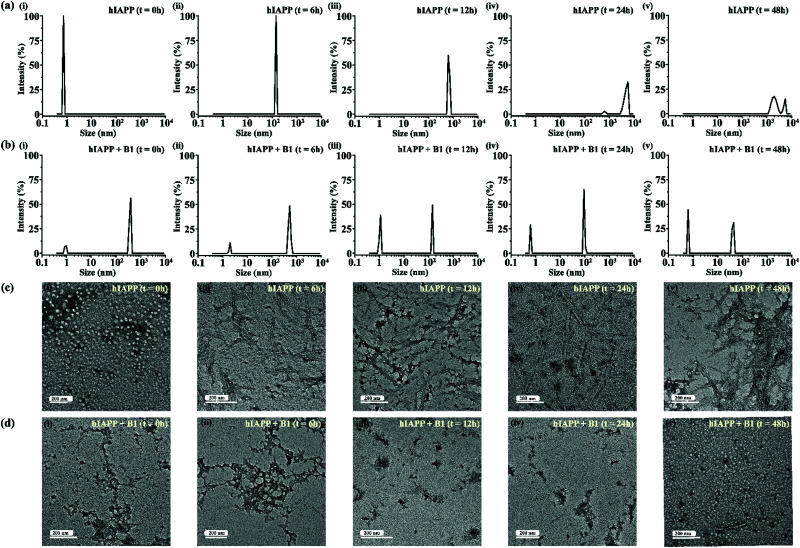
DLS results showing the size distribution of hIAPP (40 μM) alone (a) and in presence of 2-fold molar ratio of **B1** (b) at 0 h (i), 6 h (ii), 12 h (iii), 24 h (iv) and 48 h (v) of incubation respectively. TEM images showing morphological change of hIAPP (40 μM) alone (c) and in presence of 2-fold molar ratio of **B1** (d) at 0 h (i), 6 h (ii), 12 h (iii), 24 h (iv) and 48 h (v) of incubation respectively. The scale bar in the TEM images indicate 200 nm.

However, disruption of fibrils by **B1** and **C** refrained them from pore formation on the LUVs significantly, as the increment of corresponding fluorescence intensity (sample 4, magenta, and sample 5 olive, Fig. 5(b and c)) was as low as that of the untreated LUVs (sample 1, black, Fig. 5(b and c)). Hence, these results indicate that the disrupted fibrillar assembly of hIAPP by **B1** and **C** does not damage the cell membrane, which may be related to their non-toxicity.

### Preliminary investigation of the mode of inhibition of aggregation of hIAPP

For gaining insight into the mechanism of inhibition of hIAPP aggregation by breaker peptide (**B1**), we investigated the size distribution and change in morphology of hIAPP alone and the presence of **B1** using Dynamic Light Scattering (DLS) experiment and TEM, respectively.^30,31^ Moreover, we carried out dye leakage assay using freshly prepared carboxyfluorescein dye entrapped LUVs to investigate the membrane-disrupting nature of the species present during fibrillization of hIAPP and its inhibition by **B1**.^32^ For the entire study, hIAPP (40 μM) alone and in the presence of a 2-fold molar ratio of **B1** (80 μM) were incubated at pH 7.4 and 37 °C for 48 h.

In the DLS study, the size distribution in terms of hydrodynamic diameter (*d*) of hIAPP particle was observed to change in ascending order from 1 nm to several micrometers, broadening the d value. At 0 h, hIAPP exhibited a size distribution at around 1 nm, which indicated the formation of monomeric species (Fig. 5(a)-(i)). Oligomeric intermediates with size distribution centered at ∼100 nm (Fig. 5(a)-(ii)) was noted after 6 h. Further incubation up to 48 h resulted in the gradual increase in the size of hIAPP particles ranging from 100–10 000 nm (Fig. 5(a)-(iii–v)). In contrast, when hIAPP was incubated in the presence of **B1**, the fibrillization process was modulated, as in 0 h, two types of species, one having size distribution centered at 100–1000 nm and the other with a reduced size distribution at around 1 nm were observed instantly (Fig. 5(b)-(i)). The appearance of a size distribution at 100–1000 nm indicates that hIAPP assembles with **B1** to generate some specific aggregated species, altering the native aggregation pathway of hIAPP. As **B1** restricted the native fibrillation of hIAPP, the initial aggregated species gradually reduced in size, evident from the substantial change in the size distribution with time (Fig. 5(b)-(ii–v)).

Further, a transitional growth of hIAPP from smaller to longer fibrils, finally leading to a more extensive fibrillar network, was observed under TEM. At 0h of incubation, hIAPP existed entirely in dot-like form. It slowly developed intermediate smaller fibrillar species at 6–12 h, followed by the formation of the mature fibrillar network after 24 h (Fig. 5(c)-(i–v)). On the contrary, in the presence of **B1**, the entire process of hIAPP fibrillization was modulated. Some aggregated species of altered morphology along with a minute amount of fibrils were initially generated upon binding of hIAPP to **B1**. The fibrillar assembly was not observed anymore after six hours, gradually transforming the non-fibrillar aggregates into smaller dot-like species (Fig. 5(d)-(i–v)).

**Fig. 6:**
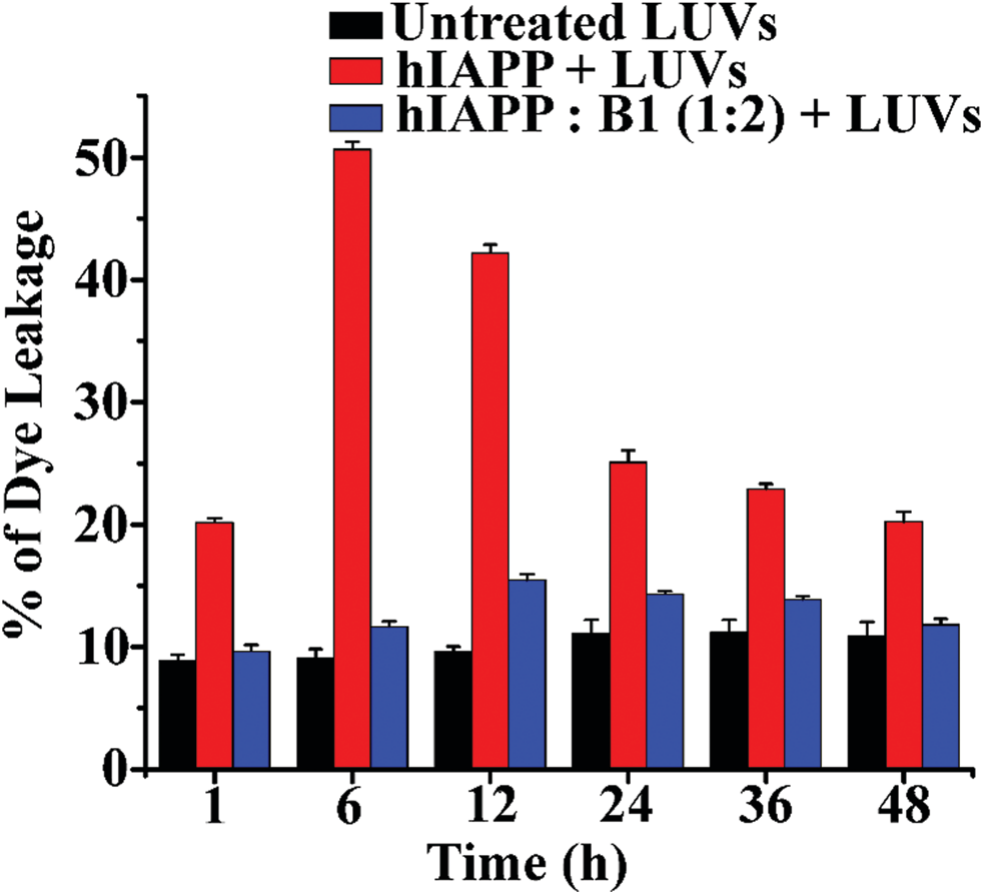
The emission of carboxyfluorescein dye showing the effect of hIAPP on the large Unilamellar vesicles (LUVs) with time and % of dye leakage during the inhibition process in the absence and presence of **B1** with hIAPP from 1 to 48 h.

In the dye leakage assay, hIAPP in the absence and presence of **B1** were incubated for 0, 1, 6, 12, 24, 36, and 48 h in PBS and then added to LUVs to obtain a final concentration of 50 μM of the lipids. We observed the most significant leakage of carboxyfluorescein dye from the 6h incubated hIAPP sample (red, Fig. 6). However, the leakage decreased gradually upon increasing the incubation time of hIAPP from 12 to 48 h, indicating the higher toxicity arising from the oligomeric species generated at around 6 h and lower toxicity of the mature fibrils. In contrast, the dye leakage by hIAPP co-incubated with **B1** (blue, Fig. 6) was not significant as the corresponding fluorescence intensity appeared as low as the untreated LUVs (black, Fig. 6).

Therefore, from the observation of size distribution and morphology, it can be inferred that binding of the breaker peptide (**B1**) with hIAPP triggers the generation of non-fibrillar aggregates, which prevents the growth of hIAPP oligomers associated with amyloid fibrils.^30,31^ Also, it was observed in the dye leakage assay that the aggregates that appeared in the presence of **B1** were not membrane damaging, thus probably non-toxic. These results are in strong correlation with the results obtained from the other biophysical studies of inhibition and disruption. Hence effective binding of the breaker peptide, **B1**, might influence the anti-amyloidogenic processing of hIAPP; however, further investigations are required to understand the mechanism correctly.

### Cell toxicity assay

**Fig. 7:**
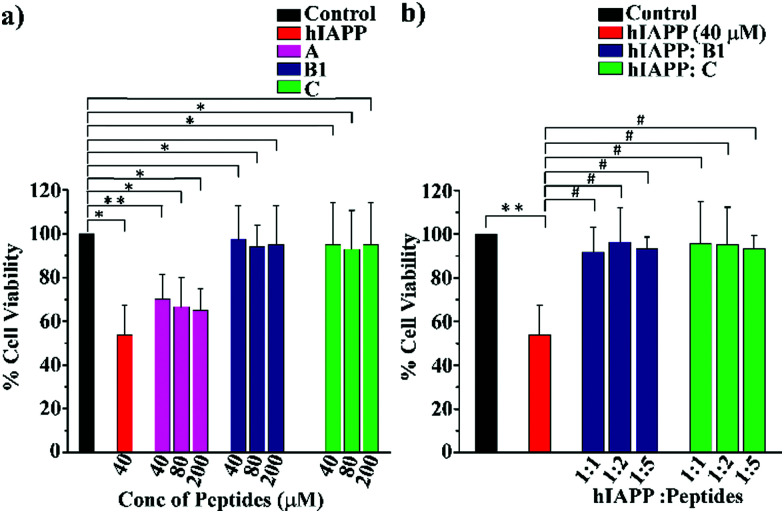
(a) RIN-5F cells viability of hIAPP and different concentration of the synthesized peptides as determined by MTT assay. (b) Cell viability of RIN-5F cells treated with hIAPP in the presence and absence of breaker peptides, **B1** and **C** (**P ≤ 0.01, *P ≤ 0.5 *vs.* control, ^#^*P* ≤ 0.5 *vs.* hIAPP treated cell).

To examine whether our synthesized peptidomimetics itself are toxic to the mammalian cell, we have tested their effects on RIN-5F (rat pancreatic cells) in culture and ascertained the cell viability using MTT assay.^31^ It is evident from the MTT assay that **B1** and **C** showed more than 80% cell viability, whereas **A** showed approximately 60% viability at 40, 80, and 200 μM concentrations (Fig. 7(a)). On the other hand, significant cytotoxicity of 50% was observed for hIAPP (40 μM) when incubated alone. Further, the same study was carried out to investigate the hIAPP induced toxicity effect of the breaker peptides (**B1** and **C**) in different molar ratios to hIAPP. The presence of the breaker peptides in the molar ratios of 1 : 1, 1 : 2, and 1 : 5 (hIAPP : peptide) rescued the cells up to more than 85% (Fig. 7(b)). Thus, from the present assay, it can be inferred that the peptidomimetics **B1** and **C** were non-toxic to the mammalian cells and found to protect cells from hIAPP-induced cytotoxicity.

## Conclusions

From the earlier reports, it was evident that insertion of a conformationally restricted element in the peptide backbone gained outstanding efficiency towards the inhibition or disruption of fibril of amyloidogenic peptides, and few of them are in clinical trials.^33–36^ We previously demonstrated that the insertion of **Ant** into a small peptide sequence inhibited hIAPP aggregation in a dose of 10-fold molar excess.^12^ Moreover, instead of **Ant**, proline and other turn-mimetic molecules could be inserted into the peptide backbone as an inhibitor of aggregation. However, **Ant** mutated peptidomimetics are usually preferred due to two significant factors. Firstly, the π-stacking ability of the aromatic moiety of **Ant** confers structural rigidity to the resulting peptidomimetics, and secondly, being a non-coded amino acid enhances the proteolytic stability.^12,13^

Here, we have demonstrated the superiority of **Ant** mutated hIAPP_8-37_ at different positions (**B1** and **B2**) over its smaller **Ant** mutant variant (**C**) for modulating aggregation of hIAPP. The designed peptidomimetics, **B1**, **B2**, and **C** were non-amyloidogenic. However, polypeptide **A**, which did not contain any **Ant**, showed amyloid aggregates similar to hIAPP in the same condition. Also, **B1** and **B2** were better inhibitors of amyloid formation than **C** and worked at a lower concentration. However, the position of mutation (G24X and I26X, X = Ant) did not change their ability to inhibit amyloidogenesis significantly.

Moreover, both **B1** and **C** disrupted preformed amyloid, and **B1** was more effective than **C**. Such amyloid disruption did not result in toxic smaller oligomers, as evident from LUV studies. Systematic DLS and TEM studies revealed that the aggregation pathway of hIAPP gets altered by **B1**, and oligomers thus generated do not rupture lipid membrane significantly. Most importantly, a gradual decrease in aggregate size was noted. MTT assay revealed that **Ant**-containing peptidomimetics are non-toxic to RIN-5F cells and rescue from hIAPP mediated toxicity. The improved efficiency of the longer peptidomimetics (**B1** and **B2**) may be due to their tight binding and more effective sequence recognition to the fibrillar species generated by the wild-type hIAPP.^7,13^ These peptidomimetics are highly efficient aggregation inhibitors and disrupt the preformed amyloid at fewer molar ratios. Therefore, these peptidomimetics can be a lead scaffold for therapeutic design towards T2D. Most importantly, these results give hope that an **Ant** mutant of hIAPP may work similarly to Pramlintide; of course, further studies are required to confirm it.

## Author contributions

Sourav Kalita, Sujan Kalita, A. P. and B. M. contributed to the design and/or execution of biochemical experiments. M. S. and Sachin Kumar designed and performed cell based experiments. All authors contributed to write the manuscript. B. M. managed funding, conceived and supervised the project.

## Conflicts of interest

There are no conflicts to declare.

## Supplementary Material

CB-002-D0CB00178C-s001
