# Supplementary material for: Site-specific single point mutation by anthranilic acid in hIAPP8–37 enhances anti-amyloidogenic activity
Source: RSC Chem Biol. 2021 Jan 15;2(1):266–73. doi: 10.1039/d0cb00178c (PMC8341151; doi:10.1039/d0cb00178c)
Supplement: CB-002-D0CB00178C-s001 [file CB-002-D0CB00178C-s001.pdf]

Electronic Supplementary Material (ESI) for RSC Chemical Biology.
This journal is © The Royal Society of Chemistry 2021

Electronic Supplementary Information (ESI)

Site-Specific Single Point Mutation by Anthranilic Acid in $hIAPP_{8-37}$ Enhances Anti-Amyloidogenic Activity

Sourav Kalita[a], Sujan Kalita[a], Ashim Paul[a], Manisha Shah[b], Sachin Kumar[b] and Bhubaneswar Mandal[a]*

[a]Laboratory of Peptide and Amyloid Research, Department of Chemistry, Indian Institute of Technology, Guwahati, Assam-781039, India.

[b]Department of Biosciences and Bioengineering, Indian Institute of Technology, Guwahati, Assam-781039, India.

Email: bmandal@iitg.ac.in

Contents: **Page No**

1. Characterization of the synthesized peptidomimetics by RP-HPLC and MALDI-TOF Mass spectrometry S2-S5
2. Materials and Methods S6-S8
3. Instrumentation S9-S13
4. LUVs preparation and carboxyfluorescein entrapment S14
5. Cell toxicity assay S15
6. Inhibition of amyloid of hIAPP aggregation by different doses of the peptidomimetics S16
7. Disruption of preformed amyloid of hIAPP by different doses of the peptidomimetics S17-S18
8. References S19

Characterization data of the synthesized peptidomimetics by HPLC and MALDI Mass spectrometry:

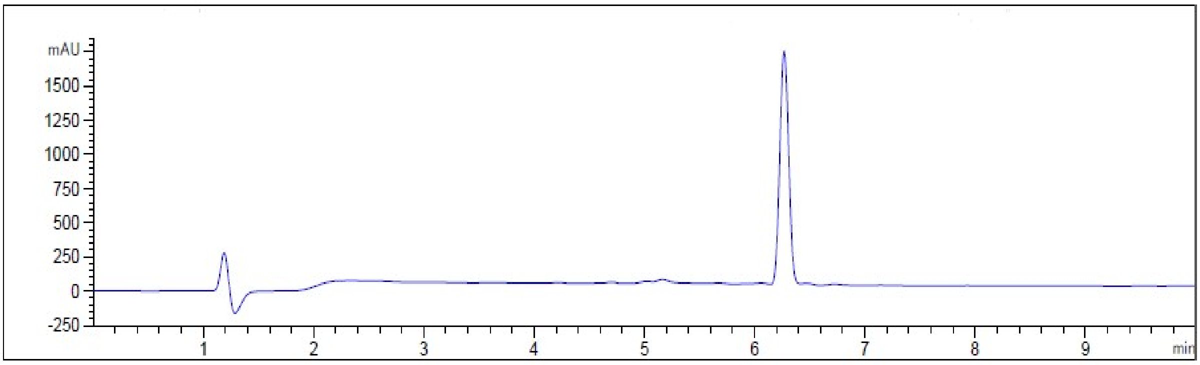

Fig S1: RP-HPLC profile of purified peptide **A**

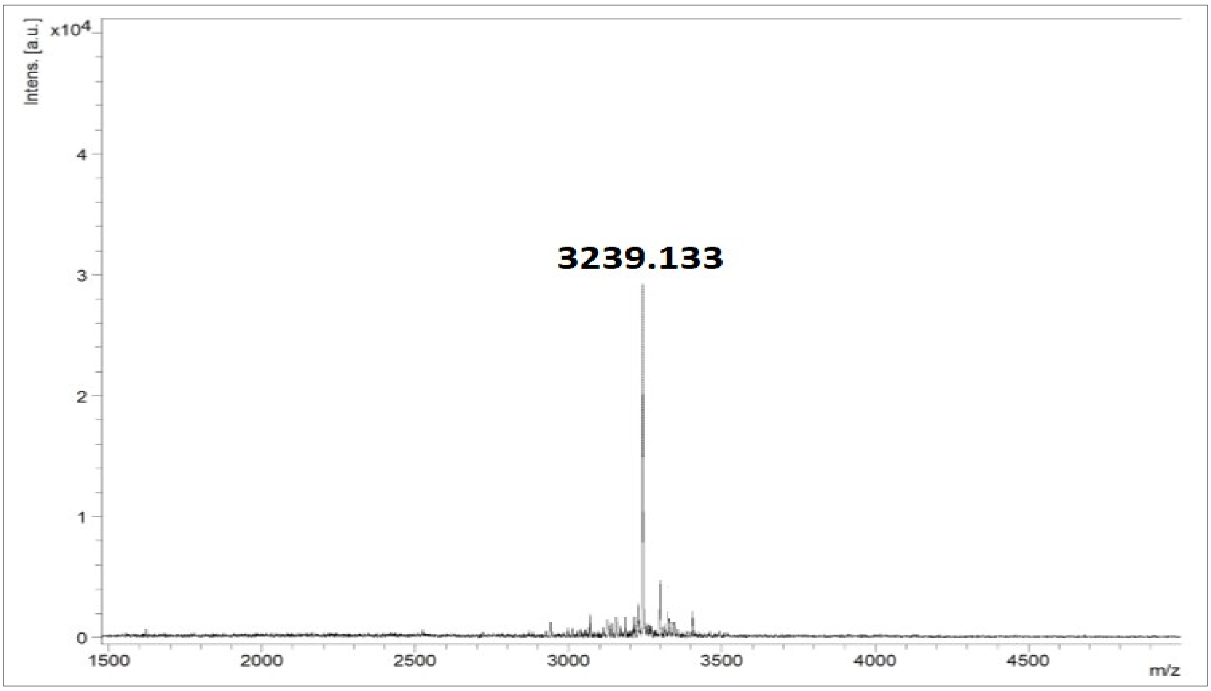

Fig S2: MALDI-TOF mass spectrum of peptide **A**. Calculated mass for $C_{140}H_{219}N_{43}O_{46}$ is 3238.61, observed mass is 3239.133 $[M+H]^+$.

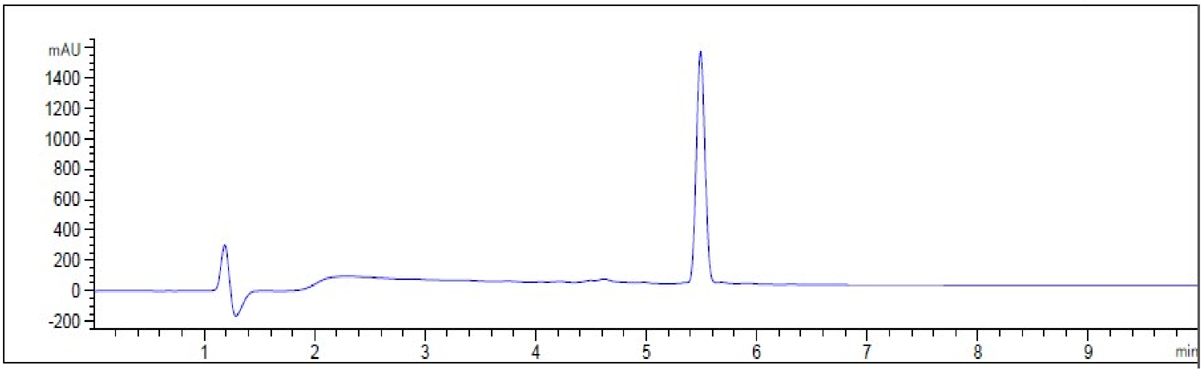

Fig S3: RP-HPLC profile of purified peptide **B1**

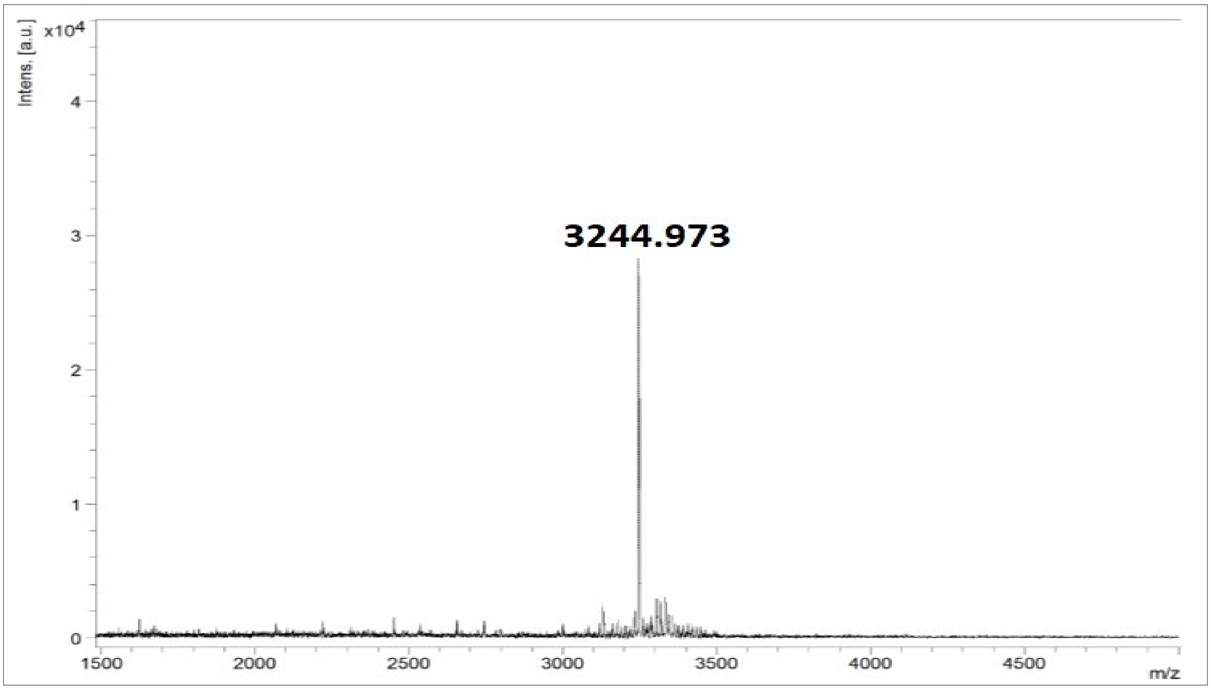

Fig S4: MALDI-TOF mass spectrum of peptide **B1**. Calculated mass for $C_{143}H_{218}N_{42}O_{45}$ is 3243.606, observed 3244.973 $[M+H]^+$:

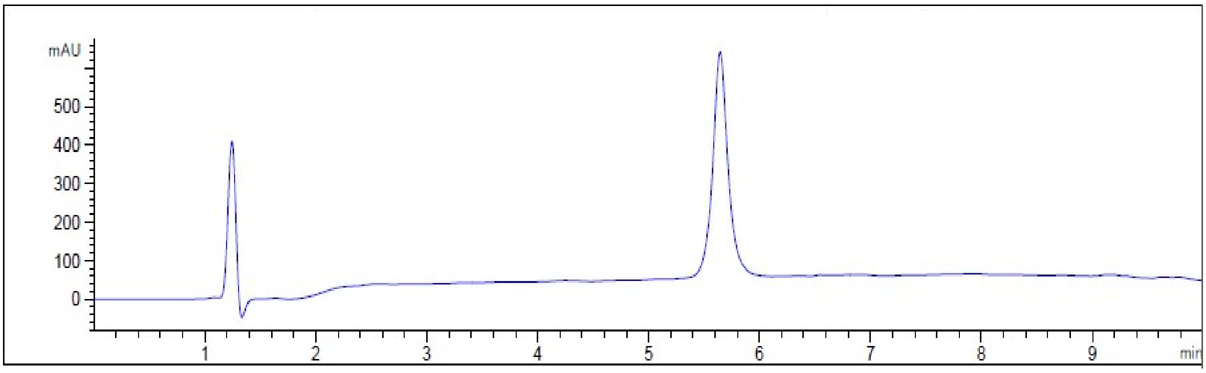

Fig S5: RP-HPLC profile of purified peptide **B2**

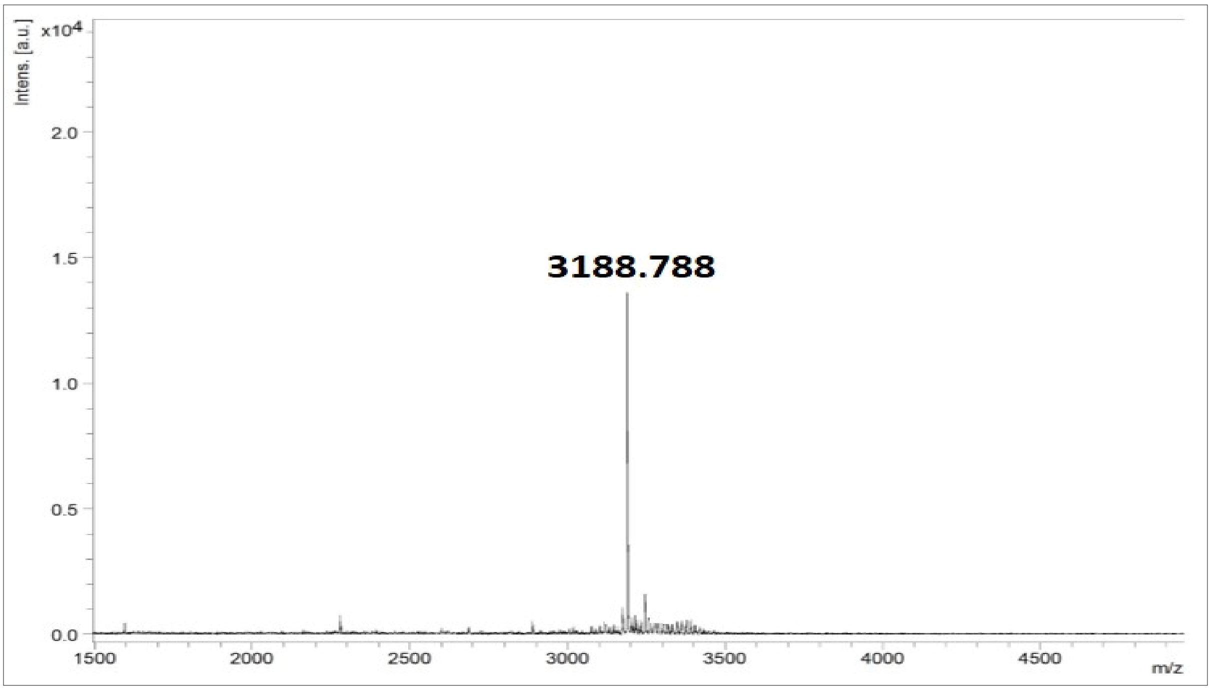

Fig S6: MALDI-TOF Mass spectrum of peptide **B2**. Calculated mass for $C_{139}H_{210}N_{42}O_{45}$ is 3187.543, observed 3188.788 $[M+H]^+$

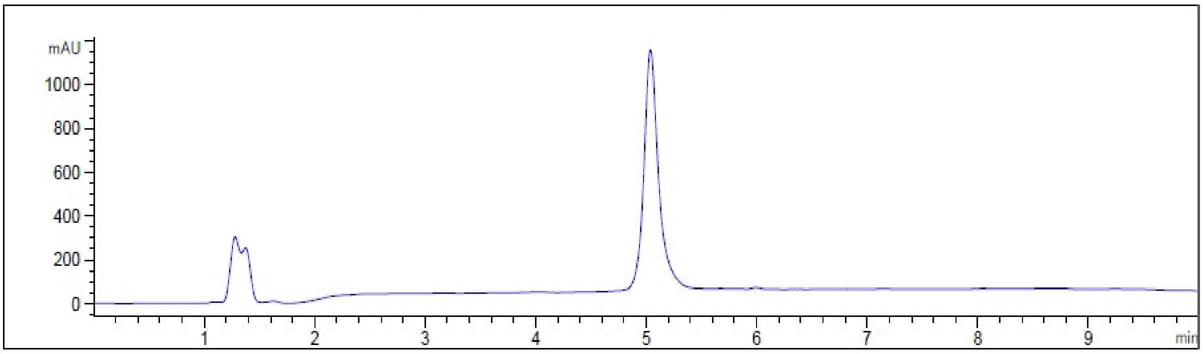

Fig S7: RP-HPLC profile of purified peptide **C**

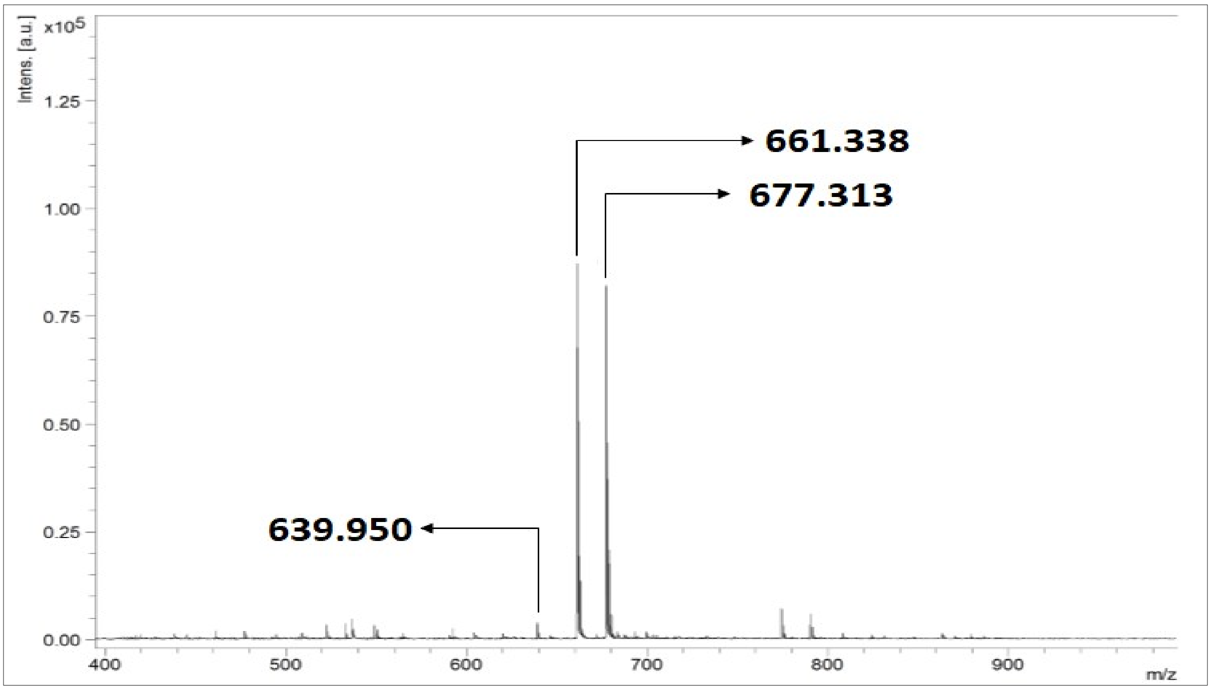

Fig S8: MALDI-TOF mass spectrum of peptide **C**. Calculated mass for $C_{31}H_{42}N_8O_7$ is 638.317, observed 639.950 $[M+H]^+$, 661.338 $[M+Na]^+$, 677.313 $[M+K]^+$

Material and Methods:

Reagents and solvents

Rink amide MBHA resin (loading 0.7mmol/g), human Islet Amyloid Polypeptide (hIAPP), all Fmoc amino acids with the specific side-chain protecting groups i.e., t-Butyl for Glutamic acid and Pbf for Arginine, and BOP [(Benzotriazole-1-yloxy) tris (dimethylamino) phosphoniumphosphate] were purchased from GL Biochem (Shanghai). Ethyl 2-cyano-2-(2-nitrobenzene-sulphonyl oxyimino) acetate known as *o*-Nosyloxy was prepared in our laboratory. DIPEA, Fmoc-Ant-OH, and 5(6)-Carboxyfluorescein were purchased from Sigma. Ferric Chloride ($FeCl_3$) was purchased from Alfa-Aeser. Cholesterol (99%), acetic anhydride (Ac_2O) of synthetic grade, N-methyl imidazole (NMI) of extra pure grade, DMSO of synthetic grade and TFA of extra pure grade were purchased from SRL (India). 1,2-Dipalmitoyl-*sn*-glycero-3-phosphocholine (DPPC), Ganglioside GM1 were purchased from Avanti Polar Lipid, Inc. Acetonitrile of HPLC grade, DCM and DMF of extra pure grade were obtained from Merck (India). Milli-Q water was used from a departmental laboratory with a filter of 18.2 Ω.

Peptide synthesis

All the designed peptides were synthesized by standard solid-phase peptide synthesis (SPPS) method using Fmoc/t-Bu strategy on Rink Amide MBHA resin as the solid support. (Scheme 1).[1] The resin was swollen in DCM for 3h, followed by DMF for 1h. Fmoc deprotection was carried out using 20% piperidine in DMF for 21 mins (7 × 3). Coupling at each step was carried out using 2 eq of Fmoc-amino acids, 2.5 eq of *o*-Nosyloxy as a coupling reagent, and 5 eq of DIPEA as a base. Each amino acid coupling was monitored by Kaiser‘s test and if found incomplete, the coupling steps were repeated for 2nd time followed by capping with Ac_2O (2

equiv) and NMI (3 equiv) in DCM for 1.5 h. More emphasis was given on the coupling next to Anthranilic acid, as it is very difficult due to steric effects. Coupling was repeated for the entire sequence maintaining the same protocol. After completion of peptide synthesis, the final peptide was cleaved from the resin using 80% TFA, 15% DCM and 5% H_2O for 3h. The crude peptides were precipitated in cold diethyl ether, followed by centrifugation and then purified by semi-preparative HPLC using H_2O and ACN as a binary solvent system. The purified peptides were characterized by HPLC and MALDI mass spectrometry.

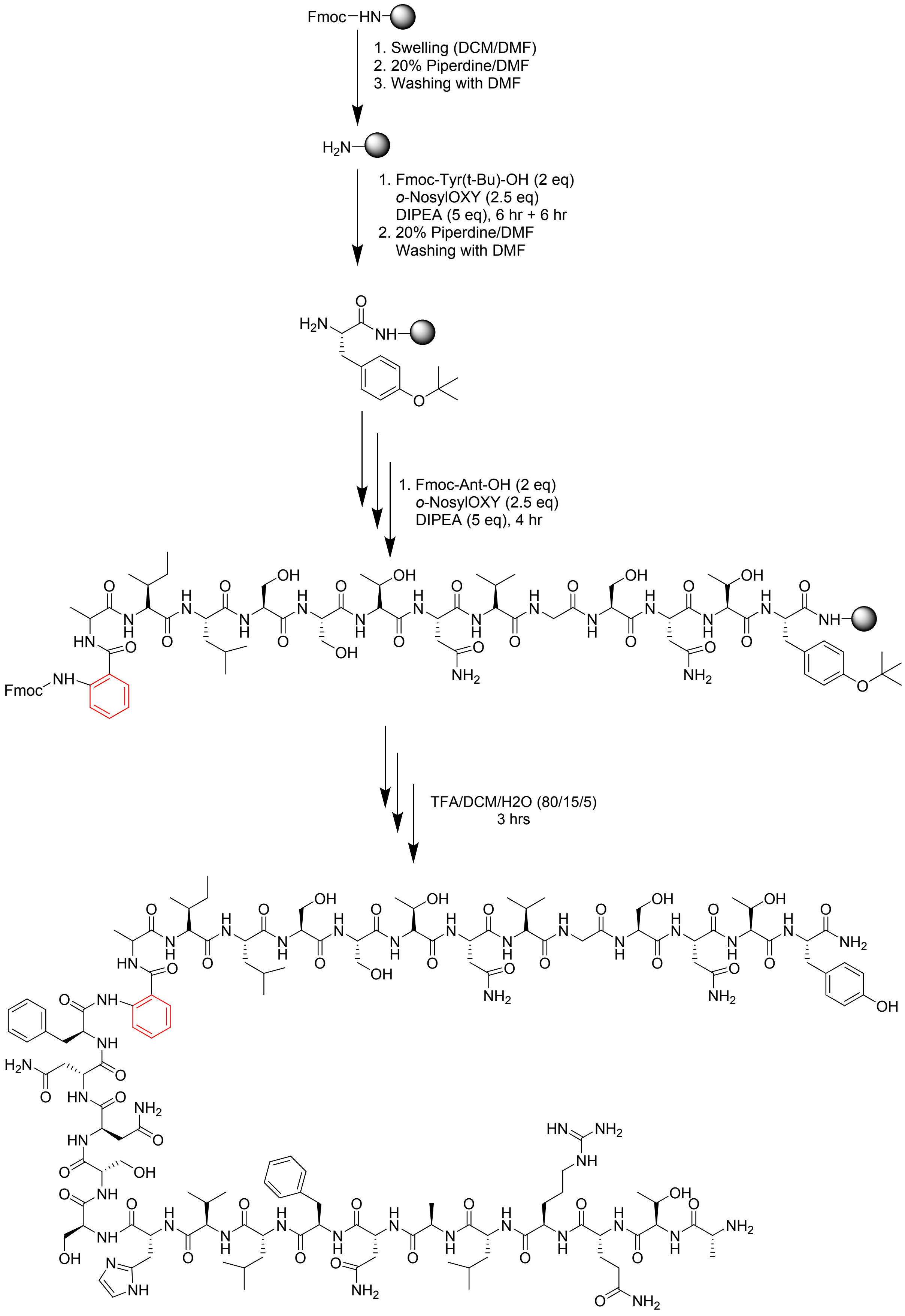

Scheme S1: The synthetic scheme of peptide **B1**

Instrumentations:

High-Performance Liquid chromatography and MALDI-TOF mass spectrometry

The crude peptides were purified by RP-HPLC (Thermo Scientific) after dissolving in acetonitrile/water mixture using a semi-preparative C18-μ Bondapak column (250 × 10 mm dimension, 12 μM particle size, 175 Å pore size) at a flow rate of 5 mL/ min. A binary solvent system was used containing solvent A (0.1 % TFA in H_2O) and solvent B (0.1 % TFA in CH_3CN). The dual detector wavelength was set at 214nm and 254 nm. A linear gradient was used for purification of the peptides of 5−100 % CH_3CN for 18 min followed by 100% CH_3CN till 20 min, in a total of 20 min of run time. The Purity of the peptides were further confirmed using Agilent analytical HPLC system with Agilent C18 analytical column at a flow rate of 1 ml/min, with a linear gradient of 5-95 % CH_3CN up to 8 minutes, 95% CH_3CN until 9 mins followed by 5% CH_3CN up to 10 mins, in a total run time of 10 min. Further, the purified peptide solution was mixed with CHCA (α-cyano-hydroxycinnamic acid) matrix in a 1:1 ratio and analyzed by MALDI-TOF using Bruker Daltonix Flex Analysis.

hIAPP Sample preparation.[2]

The Amylin or $hIAPP_{(1-37)}$ (~96% pure) was purchased from GL Biochem Shanghai, China, and used directly without further purification. 3.2 mg of $hIAPP_{(1-37)}$ was dissolved in 20 μL of HFIP to obtain disaggregated hIAPP and HFIP was evaporated by purging nitrogen gas. This process was repeated twice. To this, 1.3 ml of 50 mM PBS of pH 7.4, containing 3% DMSO was added to the disaggregated material followed by sonication and vortex to obtain a transparent solution. From the solution, 50 μl of each aliquot was transferred into 26 equal portions. For the native hIAPP solution, the final volume was made up to 800 μl with PBS of pH 7.4 to obtain a 40 μM concentration. For the other sets, the required amount of peptides were added with the hIAPP solution in terms of their doses, and the final volume was made up to 800 μl to maintain the same concentration of the aggregating peptide.

Thioflavin T Fluorescence Assay[3]

Thioflavin T (ThT) was purchased from Sigma-Aldrich and prepared a stock solution of 50µM in PBS (50mM, pH 7.4) and stored at 4 °C for further uses. All the synthesized peptides and hIAPP (alone and in some cases mixed with breaker peptides in different molar ratios) were dissolved in 50 mM PBS of pH 7.4 with 3% DMSO to obtain a desired stock solution of 40 µM and incubated them at 37 °C on a water bath.

To perform the fluorescence assay, 40µL of peptide samples were taken out from the stock solution, mixed with 200µL of ThT solution (50µM); final volume was made up to 400µL with the addition of 160µL PBS (50 mM, pH 7.4). However, for the reference sample, 200 µL of PBS solution was mixed with 200µL of ThT solution. For the ThT fluorescence assay, emission was measured at 485nm fixing the excitation at 440nm, with each scan repeating three times using a slit of 5 nm on a Fluoromax-4, Horiba instrument up to 132 h/144h in a 12h interval. The data received from the software transferred to excel file and using these data graphs were plotted using OriginPro 8 software. For each measurement, we have prepared two sets of samples and three readings were scanned separately for each set. Thus, an average of six readings was taken and plotted as F/F_0 along the y-axis (where, F= average fluorescence value of the samples at time t, F_0= fluorescence value of the reference in absence of sample at time t) against time (h) along the x-axis with observed standard deviation set as y-error.

From the same study, relative % of amyloid present in the sample was also calculated at the highest time of incubation, i.e. at 132 h for the inhibition study and 144 h for the disruption study using the following formula.

$$\%\ of\ amyloid = \frac{(Observed\ fluorescence\ in\ presence\ of\ the\ peptide - 1)}{(Observed\ fluorescence\ in\ absence\ of\ the\ peptide - 1)} \times 100\ \%$$

Where, 1 was considered as a normalization factor, as the minimum value of F/F_0 is 1.

Transmission Electron Microscopy (TEM)[4]

The morphology of the native peptides and hIAPP in the absence and presence of the inhibitors were analyzed on JEOL (Model: JEM-2100 F Field Emission Electron Microscope) at 200 KV. After 5 days of incubation at physiological condition, 10µL aliquot of each from the stock peptide solution was added separately over the dark side of the carbon-coated copper grid and air-dried for 2 minutes. Thereafter, the samples were negatively stained by the addition of 2% uranyl acetate solution (10µL) and were allowed to float for 2 min And the excess solution was removed using blotting paper. The sample was dried at room temperature and was kept in a desiccator before taking TEM analysis. Each TEM observation was accompanied by at least three different locations and typical images were reported.

Congo-Red Stained Birefringence[4]

Congo-red was purchased from Sigma-Aldrich and added in a saturated amount to a preformed solution of 80% EtOH and 20% water containing the saturating amount of NaCl. The saturated solution was stirred and filtered through a filter paper to obtain the final working solution. A 10µL aliquot from the 5 days incubating peptide solution was placed over a microscopic glass slide and kept for 6h for air dry completely. Thereafter 10 µL of the filtered Congo red solution was added over the samples and air-dried at room temperature for 6h. Finally, the prepared samples were examined under a Leica ICC50 HD polarizable microscope.

Circular dichroism (CD)[5]

To perform the CD study, stock solutions after incubation of 5 days, the native peptides (40µM) and hIAPP (40µM) in absence and presence of 20µM, 40µM, and 80µM peptides were recorded without further dilution. A total volume of 200µL of the peptide solutions was taken in a 1mm path length quartz cuvette and an average of three scans was accumulated for each sample. Spectra were recorded from 190nm to 260nm with 1 nm bandwidth and with a continuous 50 nm/min scanning speed on a JASCO J-1500 spectrometer. The spectrum for each sample was subtracted from the baseline in the software.

Observed ellipticity (mDeg) was converted to mean residue molar ellipticity using the following equation:

θ (deg.cm.^{2}dmol^{-1}) = Ellipticity (mDeg).106/Path length (mm).[Protein]. N

Where [Protein] represents the concentration of the peptide solution in µM and N represents the no of the amide bonds in the peptide sequence.

Fourier transformation infra-red (FT-IR)[4,6]

FT-IR spectra were recorded after 5 days of incubation of the peptide solutions at 37 °C. An aliquot of 20 µL was taken out from the stock peptide solution, mixed with KBr, dried the sample overnight and a pellet was prepared. Initially, the background scan was recorded and subtracted from the sample scans. The text files were plotted using OriginPro 8 software.

Dynamic Light Scattering:[7]

We performed DLS measurements for the hIAPP alone and in presence of 2-fold molar ratio of at different time intervals at 25 °C using a 633 nm He-Ne laser on a Zetasizer Nano-ZS90 (Malvern Instruments). For the experiment, we prepared a stock solution of hIAPP (40 µM) in

PBS of pH 7.4 (with 3% DMSO) and then added required amount of **B1** solution to get final ratio of 1:2 (hIAPP: **B1**). Then the hIAPP alone and the mixture were incubated at 37 °C and the size distribution measurements were recorded at different time interval using 200 μL of each the samples in a cuvette. Prior to scanning, the system was equilibrated for 120 seconds. Each measurements recorded intensity percentage along with size distribution, which were an average of 10 scans. The text values were obtained directly from the Zetasizer software, plotted using OriginPro 8 software and finalized in adobe illustrator.

Large Unilamellar Vesicles (LUVs) Preparation and Carboxyfluorescein Entrapment[8,9]

For the preparation of LUV, three different lipids were used, namely DPPC, Cholesterol, and GM1 with 68:30:2 molar ratios. Before the LUVs preparation, all the lipids were taken in a clean Eppendorf and dissolved in a 2:1 ratio of chloroform and methanol to make 2 mM stock solution and the solvents were evaporated completely to form a thin lipid film by purging nitrogen gas. Thereafter, the carboxyfluorescein dye dissolved in 500 μL of 50 mM PBS of pH 7.4 to prepare a stock solution of 200 μM, followed by hydration of the lipid film in the prepared dye solution. Further, the solution was vortexed vigorously for 30 min to emulsify the lipid mixtures. Then, the glass vial was dipped into the liquid nitrogen for instant cooling and after 5 min the frozen solution was dipped into a water bath at 50-60 °C for thawing for complete entrapment of the dye. The same step was repeated five times. The resulting solution was centrifuged at 20000 rpm to remove the excess dye as the supernatant dye solution was discarded and the lipid pellet was re-hydrated with 50 mM HEPES buffer. This step was repeated 2 more times and the final lipid pellet was obtained followed by addition of 500 μL of 50 mM of PBS buffer and vortexed to get a homogenous suspension of 2 mM stock solution of the lipid. Finally, the resulting lipid solution was filtered through a 0.45 μm polycarbonate membrane to obtain the dye loaded LUVs and the dye leakage study was performed on a Fluoromax-4, Horiba instrument. The formation of LUVs was monitored by TEM.

Then, the different peptide solutions were added separately to the freshly prepared LUVs and the dye leakage study was performed. The peptide and lipid ratio was maintained as a 1:20 molar ratio, where, with the 50 μL of the peptide solutions 25 μL of the dye-loaded LUVs were added and diluted up to 1000 μL to obtain a final concentration of 50 μM for the lipid solution and 2.5 μM for hIAPP solution.

Cell Viability Assay:

To investigate the cytotoxicity of peptide, RIN-5F (rat pancreatic cells) with density of 10^5 cells/well (per 100 µl) were seeded in 96 well tissue culture plate. The RIN-5F cells were procured from National centre for cell science, Pune India. The cells were incubated for 18h in RPMI-1640 media (Gibco) along with 10% fetal bovine serum (FBS) and 1% L-glutamine, 1% penicillin/streptomycin (Gibco) at 37°C in a humidified atmosphere under 5% CO2. The media were discarded and the cells were washed with 1X phosphate buffer saline (PBS) twice. Then peptide with different concentration was added to each well and allowed to incubate for 24h. Mitochondrial activity of cells is assessed by enzymatic conversion of 3-(4,5-dimethylthiazol-2-yl)-2,5-diphenyl tetrazolium bromide dye (MTT). The MTT reagent has been prepared by dissolving 5 mg of MTT per ml of PBS. After the time intervals of 24h, 10 µl of MTT reagent was added to each well and further incubated for 3 h at 37 °C in a humidified atmosphere. After removal of media, dimethyl sulfoxide (DMSO) (100 µl) was added to dissolve the Formosan crystals. Cell viability has been determined using 96 well plate reader by recording absorbance at 570 nm. All experiments were repeated thrice, and the relative percentage of cell viability was presented as a relative percentage with respect to the untreated cells.

Inhibition of amyloid of hIAPP aggregation by different doses of the peptidomimetics:

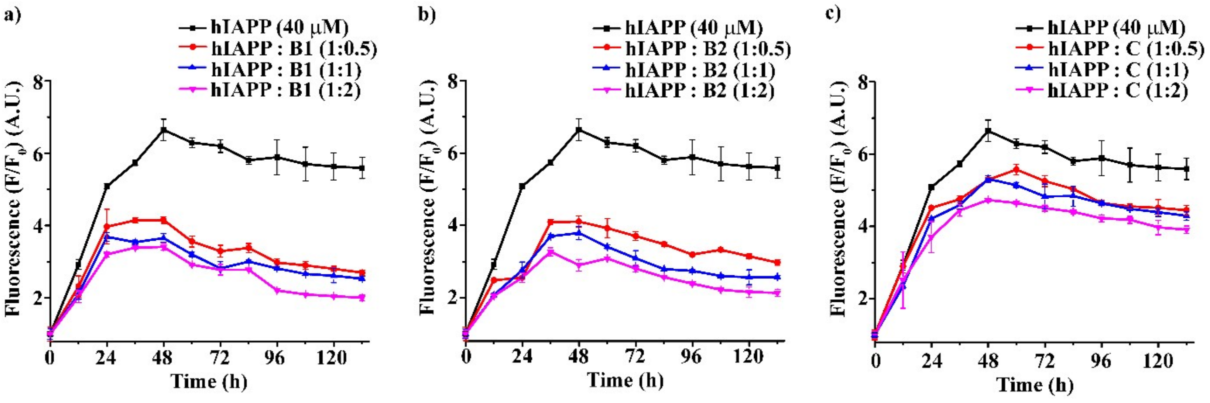

Fig S9: Time-dependent ThT fluorescence assay of hIAPP (40 µM) in the absence (black) and presence of 0.5-, 1- and 2-fold molar excess of a) **B1**, b) **B2** and c) **C**. The peptide solutions were incubated in PBS at pH 7.4 and 37 °C and studied up to 132h in 12h intervals.

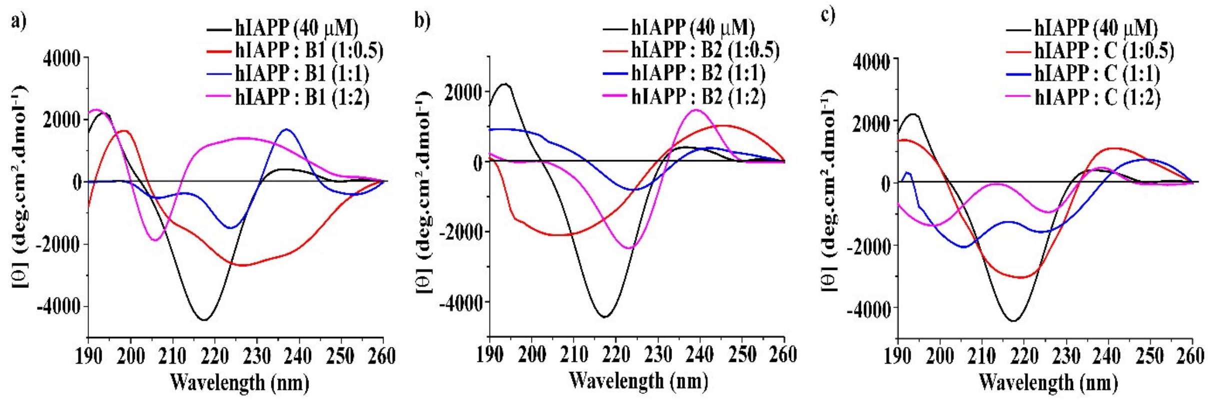

Fig S10: CD spectra of hIAPP (40 µM) in the absence (black) and presence of 0.5-, 1- and 2-fold molar excess of a) **B1**, b) **B2** and c) **C**. Spectra were recorded after 7 days of incubation in PBS of pH 7.4 at 37 °C.

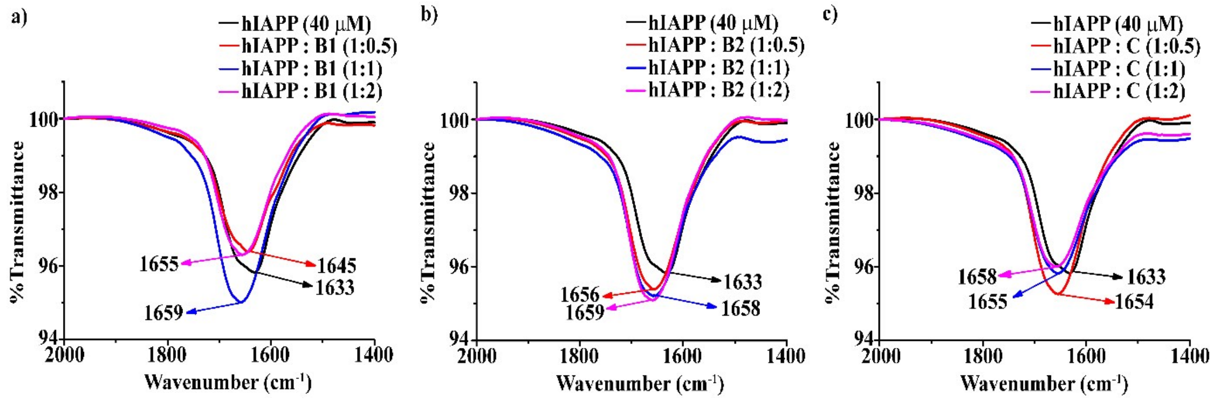

Fig S11: IR spectra of hIAPP (40 µM) in the absence (black) and presence of 0.5-, 1- and 2-fold molar excess of a) **B1**, b) **B2** and c) **C**. Spectra were recorded after 7 days of incubation in PBS of pH 7.4 at 37 °C.

Disruption of preformed amyloid of hIAPP by different doses of the peptidomimetics:

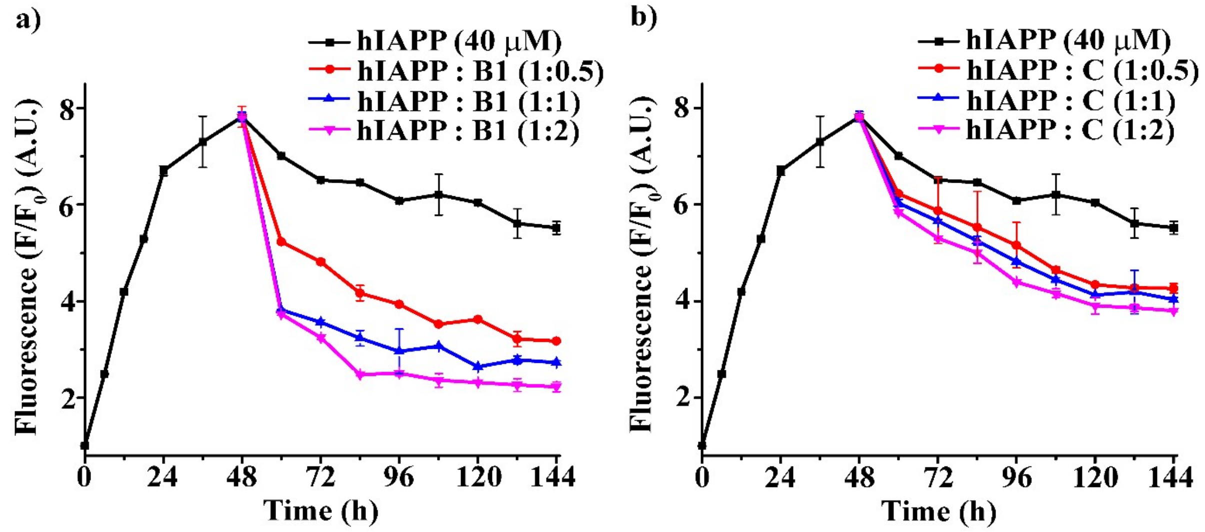

Fig S12: Time-dependent ThT fluorescence assay of hIAPP (40 μM) in the absence (black) and presence of 0.5-, 1- and 2-fold molar excess of a) **B1** and b) **C**. The peptide solutions were incubated in PBS at pH 7.4 and 37 °C and studied up to 144h in 12h intervals.

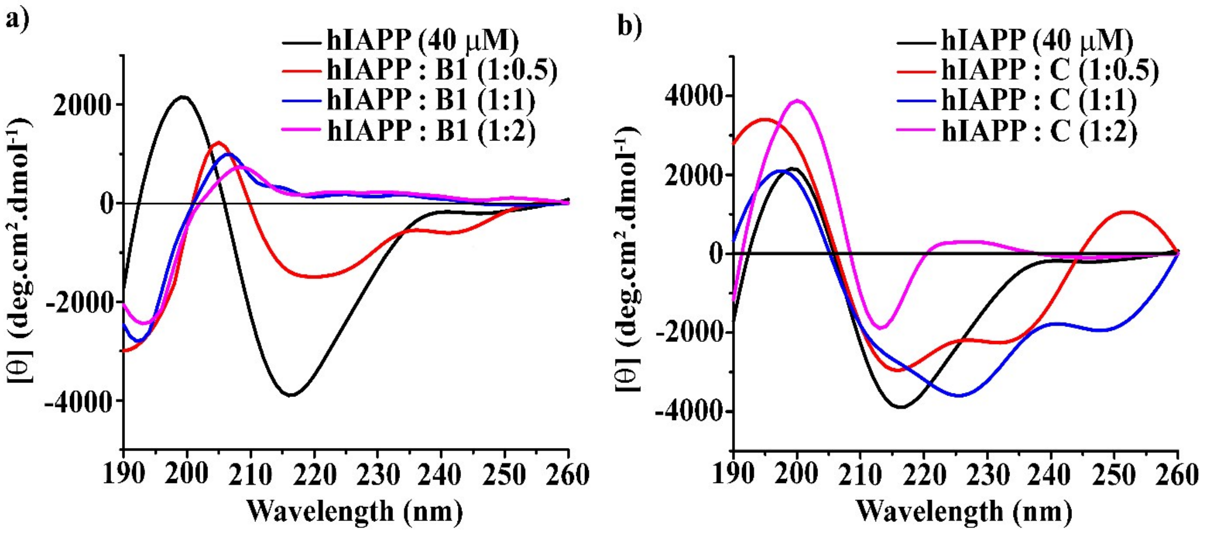

Fig S13: CD spectra of hIAPP (40 μM) in the absence (black) and presence of 0.5-, 1- and 2-fold molar excess of a) **B1** and b) **C**. Spectra were recorded after 7 days of incubation in PBS of pH 7.4 at 37 °C.

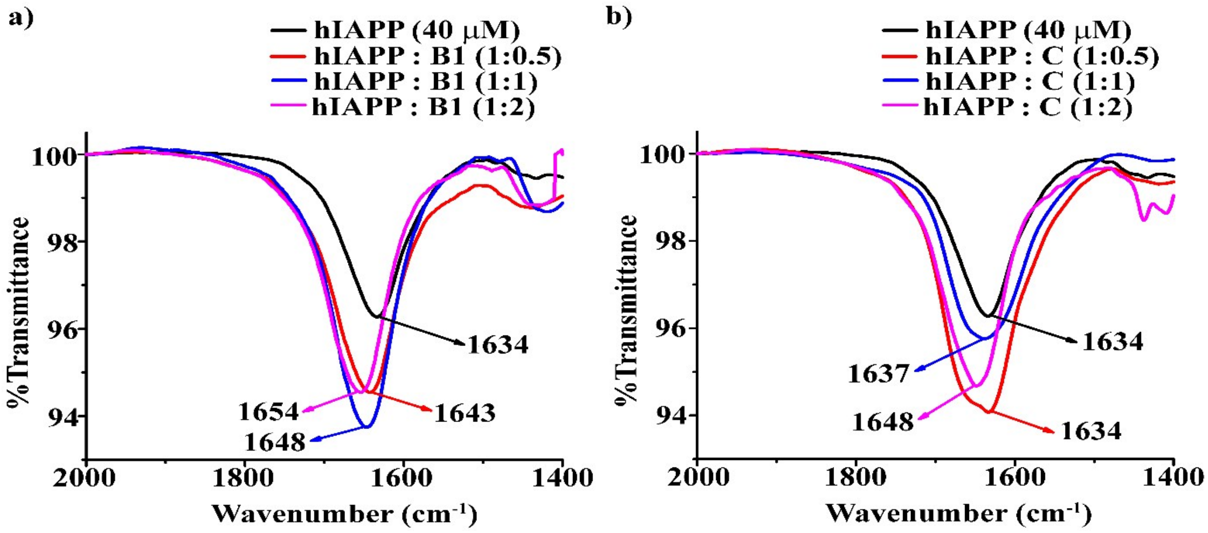

Fig S14: IR spectra of hIAPP (40 μM) in the absence (black) and presence of 0.5-, 1- and 2-fold molar excess of a) **B1** and b) **C.** Spectra were recorded after 7 days of incubation in PBS of pH 7.4 at 37 °C.

References:

1. I. Coin, M. Beyermann, M. and Bienert, *Nat. Protoc*. 2007, 2, 3247.
2. C. E. Higham, E. T. Jaikaran, P. E. Fraser, M. Gross, and A. Clark, *FEBS Letters*, 2000, 470, 55.
3. (a) H. LeVine, *Protein Sci*., 1993, 2(3) 404-410. (b) M. Biancalana and S. Koide, *Biochim. Biophys. Acta*., 2010, 1804 (7), 1405.
4. (a) M. R. Nilsson, *Methods* 2004, 34, 151. (b) P. Walsh, J. Yau, K. Simonetti and S. Sharpe, *Biochemistry*, 2009, 48(25) 5779.
5. (a) B. A. Wallace, and R. W. Janes, *Biochem Soc. Trans*., 2003, 31(3), 631. (b) S. Ma, H. Zhang, X. Dong, L. Yu, J. Zheng, and Y. Sun, *Front. Chem. Sci. Eng*., 2018, 12(2), 283.
6. B. Shivu, S. Seshadri, J. Li, K. A. Oberg, V. N. Uversky and A. L. Fink, *Biochemistry*, 2013, 52(31) 5176.
7. (a) B. Lorber, F. Fischer, M. Bailly, H. Roy and D. Kern, *Biochem and Mol. Bio*. Edu., 2012, 40, 372. (b) S. Kalita, S. Kalita, A. Paul, A. Sarkar and B. Mandal, *Chem. Sci*. 2020, 11, 4171.
8. T. L. Williams, I. J. Day and L. C. Serpell, *Langmuir*, 2010, 26, 17260.
9. M. Trakia, D. E. Warschawski, J. Cartaud, and P. F. Devaux, *Eur. Biophys. J*., 2000, 29(3), 184.
